# Post-Bleed Prognosis Beyond Hospitalization: The CLIF Consortium Acute Decompensation Score (CLIF-C AD) Predicts Six-Week Mortality After Upper Gastrointestinal Bleeding in Patients With Cirrhosis

**DOI:** 10.7759/cureus.105959

**Published:** 2026-03-27

**Authors:** Ilie Marius Ciorba, Nicoleta Craciun Ciorba, Simona M Bataga

**Affiliations:** 1 Department of Internal Medicine 1, University of Medicine, Pharmacy, Science and Technology “George Emil Palade”, Târgu Mureș, ROU; 2 Department of Medicine and Pharmacy, Institution Organizing University Doctoral Studies, University of Medicine, Pharmacy, Science and Technology “George Emil Palade”, Târgu Mureș, ROU; 3 Department of Gastroenterology, University of Medicine, Pharmacy, Science and Technology “George Emil Palade”, Târgu Mureș, ROU; 4 Department of Internal Medicine, University of Medicine, Pharmacy, Science and Technology “George Emil Palade”, Târgu Mureș, ROU

**Keywords:** 6–week mortality, acute decompensation, cirrhosis, clif–c ad, rebleeding, risk stratification, upper gastrointestinal bleeding

## Abstract

Background

In cirrhosis, outcomes after upper gastrointestinal bleeding (UGIB) extend beyond the index hospitalization. Six-week mortality is a standard endpoint for portal hypertension studies because it captures early deaths related to recurrent bleeding and post-bleed complications (infection, renal dysfunction, organ failure).

Objective

Our objective was to evaluate the CLIF Consortium Acute Decompensation score (CLIF-C AD) for post-bleed risk stratification, using six-week mortality as the prespecified primary endpoint and five-day rebleeding as a prespecified secondary endpoint, in comparison with MELD-Na (Model for End-Stage Liver Disease with sodium) and AIMS65 (albumin, international normalized ratio (INR), mental status, systolic blood pressure, age ≥65 years).

Methodology

We analyzed a retrospective cohort of 224 consecutive adults admitted with cirrhosis-associated UGIB (January 1, 2024, to December 31, 2025). No patients met the European Association for the Study of the Liver-Chronic Liver Failure (EASL-CLIF) criteria for acute on chronic liver failure (ACLF) at presentation. Risk scores were available as computed columns in the database using published definitions. Six-week vital status was obtained from regional registries and supplemented by follow-up phone calls when needed. Rebleeding at five days required recurrent hematemesis and/or melena with or without hemoglobin drop. All suspected rebleeding events underwent repeat endoscopy, and endoscopic confirmation was mandatory for classification. Discrimination was assessed with the area under the receiver operating characteristic curve (AUROC) and bootstrap 95% confidence intervals (CIs). For the secondary endpoint, we additionally evaluated an endoscopy-augmented model incorporating baseline scores, bleeding stigmata, and hemostasis modality.

Results

Six-week mortality occurred in 76 of 224 patients (33.9%), including 53 deaths during the index hospitalization and 23 additional deaths after discharge within six weeks. Five-day rebleeding occurred in 33 of 224 patients (14.7%). Among patients with esophageal varices, large varices (Paquet grade ≥3) were present in 79 of 163 (48.5%). Median CLIF-C AD was higher among non-survivors than survivors (67.7 vs. 50.5). CLIF-C AD discriminated six-week mortality with an AUROC of 0.891, comparable to MELD-Na (0.866) and AIMS65 (0.886). Optimism-corrected calibration for CLIF-C AD was near-ideal (slope 0.99, intercept -0.02), with an optimism-corrected Brier score of 0.119. Decision-curve analysis demonstrated a net benefit for CLIF-C AD over treat-all and treat-none strategies across thresholds of approximately 0.07-0.60. For five-day rebleeding, baseline score discrimination was modest, whereas an endoscopy-augmented model improved discrimination (AUROC 0.720, 95% CI 0.621-0.819). After bootstrap optimism correction, the AUROC decreased to 0.671, underscoring the need for external validation.

Conclusions

CLIF-C AD provides clinically useful post-bleed prognostication in cirrhotic UGIB when the goal is to anticipate deterioration beyond discharge. Early rebleeding prediction improves when lesion-level stigmata and endoscopic therapy are incorporated.

## Introduction

Upper gastrointestinal bleeding (UGIB) is a frequent and life-threatening trigger of acute decompensation in cirrhosis. Even when endoscopic hemostasis is achieved, early outcomes are frequently shaped by downstream complications, particularly bacterial infections, renal dysfunction, and multi-organ failure, which may occur after discharge. Portal hypertension consensus guidance, therefore, recommends six-week mortality as the benchmark endpoint for acute variceal bleeding studies because it captures the clinically relevant post-bleed window rather than only in-hospital events [1]. Risk stratification at presentation is typically performed using a mix of UGIB-specific and liver-specific scores. AIMS65 (albumin, international normalized ratio (INR), mental status, systolic blood pressure, age ≥65 years score) was derived in mixed UGIB populations and predicts inpatient mortality and resource utilization [2]. MELD-Na (Model for End-Stage Liver Disease with Sodium) incorporates hyponatremia to improve short-term mortality prediction in cirrhosis [3,4,5]. However, neither score was designed to specifically integrate the acute decompensation construct and lesion-level bleeding biology.

The CLIF Consortium Acute Decompensation score (CLIF-C AD) was developed to stratify short-term prognosis among hospitalized cirrhotic patients with acute decompensation without acute-on-chronic liver failure. It integrates age, creatinine, INR, leukocyte count, and serum sodium - variables that jointly reflect circulatory-renal dysfunction, hepatic synthetic failure, and systemic inflammation [6,7,8,9]. Those domains are central to post-bleed deterioration in cirrhosis. In our database, esophageal variceal size was encoded 1 to 4, corresponding to Paquet grades. We, therefore, evaluated CLIF-C AD for post-bleed prognosis (six-week mortality) while incorporating Paquet variceal grade as a lesion-level descriptor, particularly relevant to early rebleeding.

## Materials and methods

Study design and setting

This retrospective cohort study included consecutive adult patients admitted with cirrhosis-associated UGIB to a single tertiary referral center between January 1, 2024, and December 31, 2025. Data were extracted from electronic medical records and corroborated against endoscopy reports and transfusion logs. All patients underwent urgent upper endoscopy under a 24/7 on-call protocol.

Participants

Inclusion criteria were age ≥18 years, established or newly diagnosed cirrhosis, and acute UGIB with upper endoscopy performed during admission. Non-variceal etiologies were included (portal-hypertensive gastropathy, peptic ulcer, Mallory-Weiss tear). No patients met EASL-CLIF (European Association for the Study of the Liver-Chronic Liver Failure Consortium) criteria for acute-on-chronic liver failure (ACLF) at presentation. For patients with multiple eligible presentations, only the first eligible episode was retained.

Endpoints

The primary endpoint was six-week mortality. Six-week vital status was ascertained using regional registries and confirmed by follow-up telephone contact when registry information was incomplete. The secondary endpoint was five-day rebleeding, defined as recurrent hematemesis and/or melena with or without a hemoglobin drop and endoscopic confirmation of recurrent UGIB on chart review. Spurting and oozing were recorded as lesion activity regardless of variceal versus non-variceal etiology. When multiple bleeding lesions were present, the highest-risk stigmata category was assigned. Intensive care unit (ICU) admission during the index hospitalization is reported descriptively. The cohort was intended to reflect routine tertiary-care admissions for cirrhosis-associated UGIB rather than a preselected ICU-only population. Both previously established and newly diagnosed cirrhosis were eligible, but a standardized database field separating these subgroups was not available for reliable stratified reporting.

Clinical management

Suspected variceal bleeding was treated per institutional standard-of-care: terlipressin 1 mg every six hours for 48-72 hours and prophylactic antibiotics (ceftriaxone) according to guideline-based practice [1,4]. Endoscopic band ligation (EBL) was first-line for esophageal varices, with argon plasma coagulation (APC) or balloon tamponade used as rescue therapy when required. Self-expandable metal stents (SEMS) and transjugular intrahepatic portosystemic shunt (TIPS) were not available.

For non-variceal bleeding, patients received proton pump inhibitor therapy with an 80 mg bolus followed by 8 mg/hour infusion for 72 hours, then 40 mg twice daily. A restrictive transfusion strategy was applied: packed red blood cells (PRBC) were administered when hemoglobin was <7 g/dL or if the patient was hemodynamically unstable. Fresh frozen plasma (FFP) was administered based on coagulation dysfunction markers (INR). Platelet transfusions were not used.

Endoscopic variceal size

For esophageal varices, tokens formatted as 1-4 encode variceal size according to the Paquet classification. Where multiple grades were recorded, the maximum grade was used. Automated extraction was performed using a predefined string-matching rule, and a manual spot-check of 30 randomly selected entries containing 1-4 tokens confirmed correct mapping at the level of the stored lesion descriptor.

Risk scores and multiplicity control

All evaluated scores were available as computed columns using published definitions. Primary analyses focused on CLIF-C AD, MELD-Na, and AIMS65.

Statistical analysis

Continuous variables are presented as median (interquartile range) and categorical variables as *n* (%). Group comparisons used Mann-Whitney U tests for continuous variables and Fisher's exact or chi-square tests for categorical variables. Prespecified endpoints and primary scores were complete, and no imputation was performed. Discrimination was assessed using the area under the receiver operating characteristic curve (AUROC), with 95% CIs derived by bootstrap resampling (1,000 iterations). AUROC comparisons are presented descriptively (no formal DeLong testing) [10]. For each score, a univariable logistic regression model was fit for six-week mortality to generate predicted probabilities. Calibration was assessed using decile calibration plots and by reporting calibration intercept and slope (logistic recalibration of observed outcome on the logit of predicted risk). Overall accuracy was summarized using the Brier score [11]. Internal validation used bootstrap optimism correction (200 resamples) for AUROC, Brier score, and calibration parameters. Clinical utility was explored using decision-curve analysis (net benefit) [12] across threshold probabilities from 0.05 to 0.60 for the primary endpoint. Associations are reported as odds ratios (OR) per 1 standard deviation increase in score values. The Paquet grade was modeled per a 1-grade increase.

**Figure 1:**
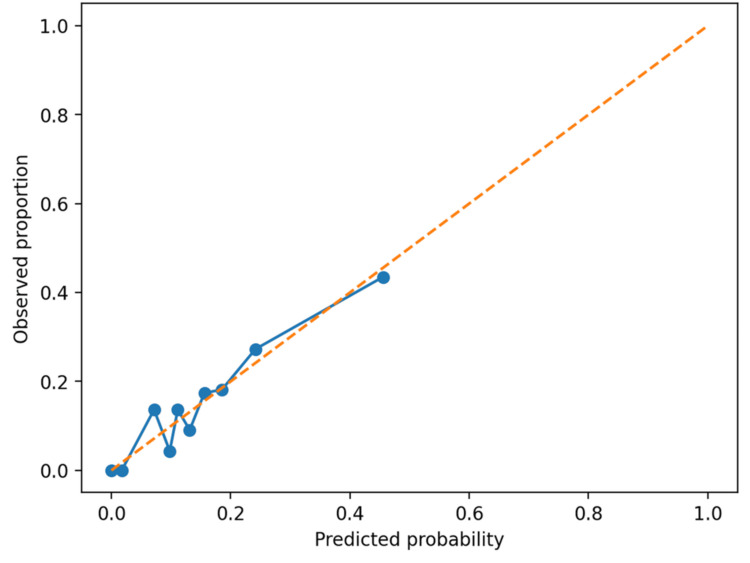
Calibration plot for the endoscopy-augmented five-day rebleeding model.

For the secondary endpoint (five-day rebleeding), ulcer lesion stigmata were classified using the Forrest classification, and variceal lesion activity was recorded as active variceal bleeding (present/absent) [13]. When multiple lesions were present, the highest-risk stigmata category was assigned. We then fitted an endoscopy-augmented model using elastic-net regularized logistic regression, incorporating baseline scores, lesion-level stigmata category, variceal etiology, and hemostasis modality. The regularization strength (C) and mixing parameter (L1 ratio) were selected by stratified 10-fold cross-validation to maximize the mean AUROC, then refitted on the full dataset. Internal validation for the rebleeding model used bootstrap optimism correction (200 resamples). Calibration was summarized using the intercept and slope and visualized with a calibration plot (Figure 1). The full coefficient set is provided in Appendix A. All analyses and figure generation were performed using Python.

Reporting follows STROBE guidance, and prediction reporting aligns with TRIPOD principles [14,15].

Ethics

This study was conducted in accordance with the Declaration of Helsinki and institutional standards for research involving human participants. The protocol was reviewed by the Mureș County Emergency Clinical Hospital Medical Ethics Commission for Clinical studies, approval number Ad 34800, and was granted approval. Because this was a retrospective analysis of routinely collected clinical data with minimal risk to participants, the requirement for informed consent was waived by the reviewing board. All data were de-identified before analysis and handled in accordance with applicable data protection policies.

## Results

The cohort included 224 patients, of whom 174 (77.7%) were male, and 194 (86.6%) had portal hypertension-related lesions on endoscopy. ICU admission occurred in 37 out of 224 patients (16.5%). Six-week mortality occurred in 76/224 (33.9%) patients, of whom 53 died during the index hospitalization, and 23 died after discharge within six weeks. Five-day rebleeding occurred in 33 (14.7%). Among patients with esophageal varices (*n* = 163), the Paquet variceal grade was available for all, with large varices (grade ≥3) present in 79 (48.5%).

Baseline characteristics stratified by six-week mortality are shown in Table 1 and by five-day rebleeding in Table 2. Compared with survivors, six-week non-survivors had higher admission severity scores (CLIF-C AD, MELD-Na, and AIMS65) and more frequent ICU admission, consistent with greater early physiologic derangement and organ dysfunction risk. Paquet grading applies only to patients with esophageal varices. Patients without esophageal varices are not graded. The study reflects a mixed ward-and-ICU population rather than an exclusively ICU cohort.

**Table 1:** Baseline characteristics stratified by six-week mortality (primary endpoint). Values are presented as median (IQR) or *n* (%). Paquet grade ≥3 denotes large varices. Statistical significance was predefined as *P* < 0.05 (two-sided). Child-Pugh class was compared as an overall three-level categorical variable using the chi-square test. CLIF-C AD, CLIF Consortium Acute Decompensation score; MELD-Na, Model for End-Stage Liver Disease with sodium; AIMS65, albumin, INR, mental status, systolic blood pressure, age ≥65 years score; INR, international normalized ratio; ICU, intensive care unit; IQR, interquartile range; PRBC, packed red blood cells; FFP, fresh frozen plasma

Characteristic	Reference range	No six-week mortality	Six-week mortality	Test statistics	P
Age (years)	-	58.0 (51.0-67.2)	62.5 (50.4-69.2)	*U* = 5,108	0.262
CLIF-C AD	-	50.5 (43.6-54.8)	67.7 (59.6-77.7)	*U* = 1,226	<0.001
MELD-Na	-	14.5 (12.0-19.0)	25.0 (20.8-34.0)	*U* = 1,506	<0.001
AIMS65	-	2.0 (0.0-2.0)	4.0 (3.0-4.0)	*U* = 1,284	<0.001
Hemoglobin (g/dL)	12.0-16.0	7.1 (6.3-8.2)	6.5 (5.6-7.0)	*U* = 7,508	<0.001
INR	0.8-1.2	1.33 (1.20-1.50)	1.91 (1.56-2.60)	*U* = 1,958	<0.001
Albumin (g/dL)	3.5-5.0	3.1 (2.8-3.4)	2.5 (2.2-2.8)	*U* = 9,455	<0.001
Total bilirubin (mg/dL)	0.2-1.2	1.5 (0.7-2.8)	3.8 (1.8-5.8)	*U* = 3,028	<0.001
Creatinine (mg/dL)	0.6-1.2	0.7 (0.6-0.9)	1.5 (0.8-2.6)	*U* = 2,536	<0.001
Sodium (mmol/L)	135-145	136 (133-139)	135 (130-140)	*U* = 6,053	0.350
Leukocytes (/µL)	4,000-10,000	6700 (4035-10375)	14205 (10178-20232)	*U* = 2,269	<0.001
Platelets (×10^3/µL)	150-400	80.0 (56.3-120.2)	91.0 (55.5-140.2)	*U* = 5,161	0.314
PRBC (units)	-	1.0 (0.0-1.0)	1.0 (0.8-2.0)	*U* = 3,984	<0.001
FFP (units)	-	0.0 (0.0-1.0)	1.0 (1.0-1.2)	*U* = 3,071	<0.001
Paquet grade ≥3 (3-4), *n* (%)	-	34 (23.0%)	55 (72.4%)	χ²(1) = 51.17	<0.001
Child-Pugh class A, *n* (%)	-	28 (18.9%)	2 (2.6%)	χ²(2) = 60.80	<0.001
Child-Pugh class B, *n* (%)	-	85 (57.4%)	15 (19.7%)		
Child-Pugh class C, *n* (%)	-	35 (23.6%)	59 (77.6%)		
Male gender, *n* (%)	-	113 (76.4%)	61 (80.3%)	χ²(1) = 0.44	0.506
Portal hypertension lesion, *n* (%)	-	130 (87.8%)	69 (90.8%)	χ²(1) = 0.44	0.507
ICU admission, *n* (%)	-	5 (3.4%)	32 (42.1%)	χ²(1) = 54.61	<0.001

**Table 2:** Baseline characteristics stratified by five-day rebleeding (secondary endpoint). Note: Values are presented as median (IQR) or *n* (%). Statistical significance was predefined as *P* < 0.05 (two-sided). The Child-Pugh class was compared as an overall three-level categorical variable using the chi-square test. CLIF-C AD, CLIF Consortium Acute Decompensation score; MELD-Na, Model for End-Stage Liver Disease with sodium; AIMS65, albumin, INR, mental status, systolic blood pressure, age ≥65 years score; ICU, intensive care unit; INR, international normalized ratio; IQR, interquartile range; PRBC, packed red blood cells; FFP, fresh frozen plasma

Characteristic	Reference range	No five-day rebleeding	Five-day rebleeding	Statistical test	P
Age (years)	-	59.0 (51.0-68.0)	58.0 (51.0-68.0)	*U* = 3,018	0.700
CLIF-C AD	-	54.0 (47.4-64.6)	53.7 (46.7-60.5)	*U* = 3,295	0.677
MELD-Na	-	18.0 (13.0-24.0)	15.0 (13.0-22.0)	*U* = 3,486	0.330
AIMS65	-	2.0 (1.0-3.0)	2.0 (1.0-3.0)	*U* = 3455	0.369
Hemoglobin (g/dL)	12.0-16.0	6.9 (6.1-8.0)	6.2 (5.8-7.4)	*U* = 3,820	0.052
INR	0.8-1.2	1.42 (1.24-1.81)	1.44 (1.21-1.78)	*U* = 3,204	0.880
Albumin (g/dL)	3.5-5.0	2.8 (2.5-3.3)	3.1 (2.5-3.4)	*U* = 2,744	0.237
Total bilirubin (mg/dL)	0.2-1.2	2.0 (0.9-4.0)	1.7 (0.7-2.8)	*U* = 3,615	0.178
Creatinine (mg/dL)	0.6-1.2	0.8 (0.7-1.4)	0.8 (0.7-1.1)	*U* = 3,329	0.607
Sodium (mmol/L)	135-145	136 (132-139)	137 (133-140)	*U* = 2,666	0.158
Leukocytes (/µL)	4,000-10,000	8750 (4885-13090)	7310 (5490-13000)	*U* = 3,224	0.835
Platelets (×10^3/µL)	150-400	80.1 (56.0-122.0)	89.0 (64.0-140.0)	*U* = 2,904	0.473
PRBC (units)	-	1.0 (0.0-1.0)	1.0 (0.0-2.0)	*U* = 2,491	0.041
FFP (units)	-	1.0 (0.0-1.0)	1.0 (0.0-1.0)	*U* = 2,924	0.464
Paquet grade ≥3 (3-4), *n* (%)	-	81 (42.4%)	8 (24.2%)	χ²(1) = 3.88	0.049
Child-Pugh class A, *n* (%)	-	25 (13.1%)	5 (15.2%)	χ²(2) = 0.51	0.776
Child-Pugh class B, *n* (%)	-	84 (44.0%)	16 (48.5%)		
Child-Pugh class C, *n* (%)	-	82 (42.9%)	12 (36.4%)		
Male gender, *n* (%)	-	149 (78.0%)	25 (75.8%)	χ²(1) = 0.08	0.774
Portal hypertension lesion, *n* (%)	-	167 (87.4%)	32 (97.0%)	Fisher	0.139
ICU admission, *n* (%)	-	34 (17.8%)	3 (9.1%)	χ²(1) = 1.55	0.213

The Child-Pugh score was calculated, and 94 (42.0%) patients were classified as class C, while class A was observed in 30 (13.4%) and class B in 100 (44.6%), further characterizing baseline cirrhosis severity [16].

Early rebleeding showed less clear separation by baseline physiologic severity scores, but was better captured by lesion-level stigmata and endoscopic therapy variables. Baseline characteristics stratified by five-day rebleeding are shown in Table 2.

**Figure 2:**
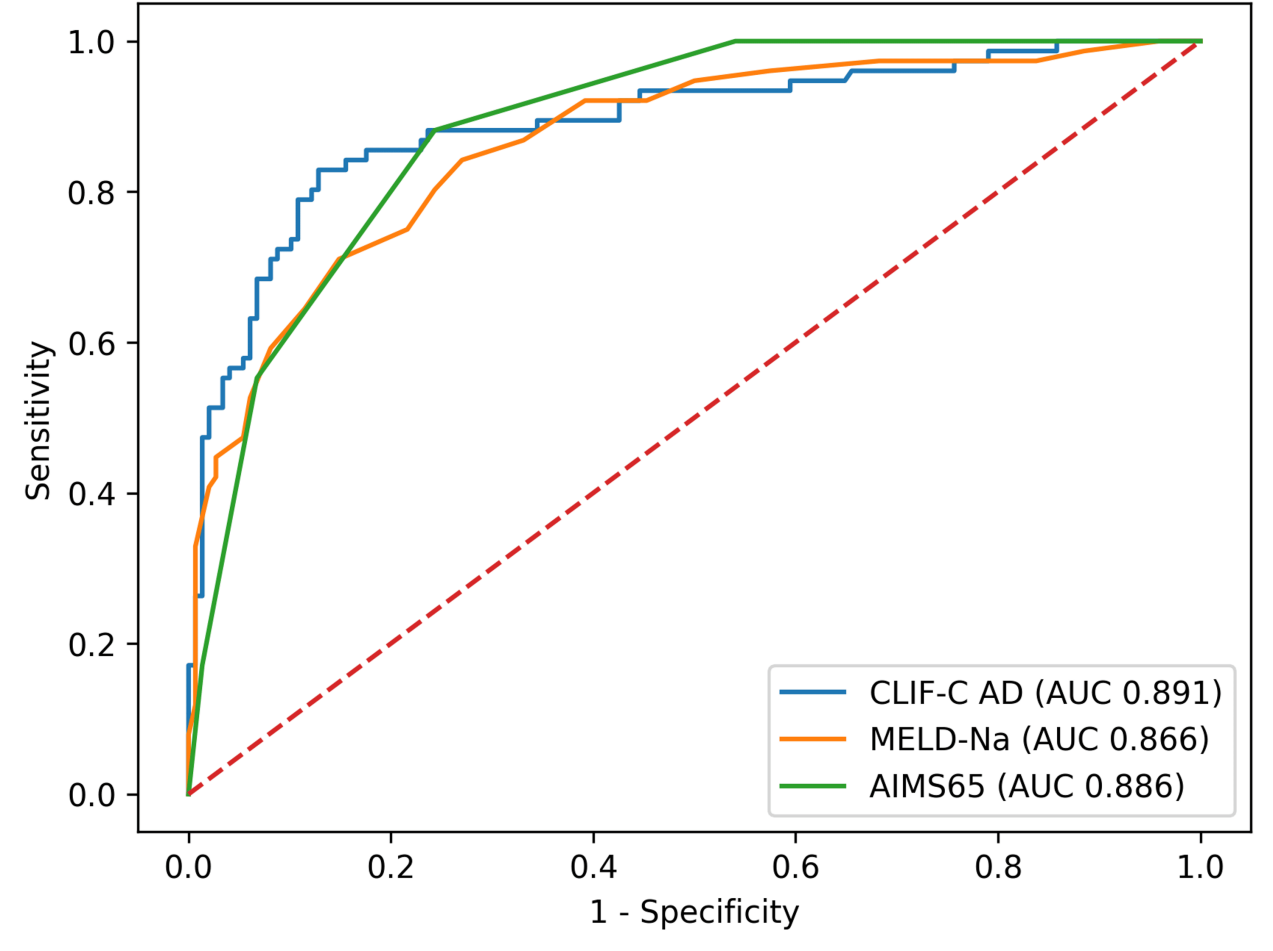
ROC curves for six-week mortality. Overlay of receiver operating characteristic curves for CLIF-C AD, MELD-Na, and AIMS65. Note: AUROC values are shown in the legend; curves are based on admission score values. ROC, receiver operating characteristic; AUROC, area under the ROC curve; CLIF-C AD, CLIF Consortium Acute Decompensation score; MELD-Na, Model for End-Stage Liver Disease with sodium; AIMS65, albumin, INR, mental status, systolic blood pressure, age ≥65 years score

Discrimination for the prespecified primary endpoint (six-week mortality) is summarized in Tables 3-4 and shown in Figure 2. All three scores showed strong discrimination with overlapping CIs. CLIF-C AD achieved AUROC 0.891 (95% CI 0.844-0.935). MELD-Na AUROC was 0.866 (95% CI 0.816-0.911) while for AIMS65, AUROC was 0.886 (95% CI 0.841-0.921). After bootstrap optimism correction, calibration was close to ideal for CLIF-C AD (intercept -0.02, slope 0.99), and overall accuracy remained acceptable (Brier 0.119). Formal pairwise DeLong comparisons for 6-week mortality showed no statistically significant differences between CLIF-C AD and MELD-Na (*P* = 0.281), CLIF-C AD and AIMS65 (*P* = 0.851), or MELD-Na and AIMS65 (*P* = 0.464).

**Table 3:** Discrimination for six-week mortality using prespecified scores. Note: AUROC 95% CIs were derived by bootstrap resampling (1,000 iterations). ORs and *P*-values were obtained from univariable logistic regression per 1 SD increase. Statistical significance was predefined as *P* < 0.05 (two-sided). AUROC, area under the receiver operating characteristic curve; CI, confidence interval; CLIF-C AD, CLIF Consortium Acute Decompensation score; MELD-Na, Model for End-Stage Liver Disease with sodium; AIMS65, albumin, INR, mental status, systolic blood pressure, age ≥65 years score

Score	AUROC	95% CI	OR (95% CI)	P
CLIF-C AD	0.891	0.844-0.935	9.68 (5.22-17.92)	<0.001
MELD-Na	0.866	0.816-0.911	6.38 (3.86-10.54)	<0.001
AIMS65	0.886	0.841-0.921	9.62 (5.23-17.69)	<0.001

**Table 4:** Discrimination, calibration, and overall accuracy for six-week mortality. AUROC values include bootstrap 95% CIs; calibration and Brier score are reported after bootstrap optimism correction. Note: Calibration intercept and slope were obtained by regressing the observed outcome on the logit of the predicted probability. Statistical significance for reported *P*-values was predefined as *P* < 0.05 (two-sided). AUROC, area under the receiver operating characteristic curve; CI, confidence interval; Brier score, mean squared error of predicted probability vs. observed outcome; CLIF-C AD, CLIF Consortium Acute Decompensation score; MELD-Na, Model for End-Stage Liver Disease with sodium; AIMS65, albumin, INR, mental status, systolic blood pressure, age ≥65 years score

Score	AUROC	95% CI	AUROC (optimism-corrected)	Brier score (optimism-corrected)	Calibration intercept	Calibration slope	OR (95% CI)	P
CLIF-C AD	0.891	0.844-0.935	0.889	0.119	-0.019	0.986	9.68 (5.22-17.92)	<0.001
MELD-Na	0.866	0.816-0.911	0.865	0.138	-0.019	0.987	6.38 (3.86-10.54)	<0.001
AIMS65	0.886	0.841-0.921	0.884	0.132	0.016	0.980	9.62 (5.23-17.69)	<0.001

To provide an interpretable measure of effect size that is comparable across different score scales, Table 5 reports odds ratios per 1 SD increase in each score. Associations persisted after minimal adjustment for age and gender, consistent with the scores’ construct validity.

**Table 5:** Logistic regression for six-week mortality: ORs per 1 SD increase. Note: Adjusted models included age and gender as covariates. ORs are expressed per 1 SD increase to support comparability across score ranges. Statistical significance was predefined as *P* < 0.05 (two-sided). OR, odds ratio; CI, confidence interval; SD, standard deviation; CLIF-C AD, CLIF Consortium Acute Decompensation score; MELD-Na, Model for End-Stage Liver Disease with sodium; AIMS65, albumin, INR, mental status, systolic blood pressure, age ≥65 years score

Score	Model	SD	OR (95% CI)	P
CLIF-C AD	Unadjusted	13.64	9.68 (5.22-17.92)	<0.001
CLIF-C AD	Adjusted (age, gender)	13.64	9.72 (5.22-18.07)	<0.001
MELD-Na	Unadjusted	8.41	6.38 (3.86-10.54)	<0.001
MELD-Na	Adjusted (age, gender)	8.41	7.72 (4.47-13.32)	<0.001
AIMS65	Unadjusted	1.51	9.62 (5.23-17.69)	<0.001
AIMS65	Adjusted (age, gender)	1.51	10.59 (5.68-19.75)	<0.001

Calibration

**Figure 3:**
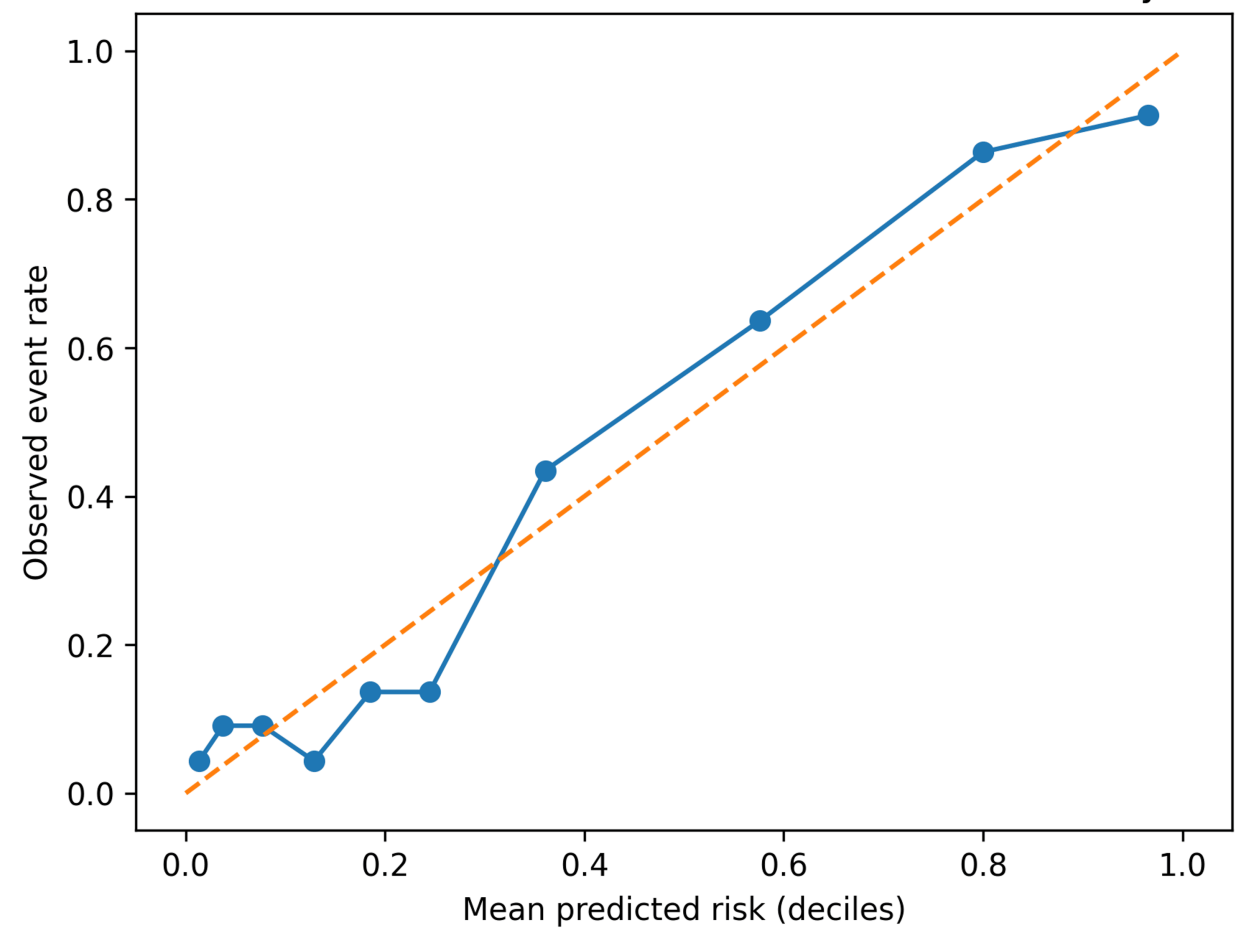
Calibration of CLIF-C AD for six–week mortality. Decile calibration plot comparing mean predicted risk with observed event rates. Note: Calibration was assessed using a logistic model with CLIF-C AD as the sole predictor. Deciles were formed from predicted probabilities. CLIF-C AD, CLIF Consortium Acute Decompensation score

Figure 3 shows decile calibration for a CLIF-C AD logistic model predicting six-week mortality. Predicted risks tracked observed risks reasonably well across the risk spectrum, supporting its use for stratification rather than precise individual risk prediction.

Secondary endpoint: early rebleeding (five days)

Baseline severity scores (CLIF-C AD, MELD-Na, and AIMS65) were similar between rebleeders and non-rebleeders (Table 6), whereas endoscopic stigmata and endoscopic therapy demonstrated greater signal for hemostasis failure. For ulcer lesions, stigmata were mapped using the Forrest classification. For variceal bleeding, we set a single category of active variceal bleeding (present/absent) and retained other variceal stigmata (white nipple sign, red wale marks). When multiple lesions were present, the highest-risk stigmata category was assigned. We then evaluated an endoscopy-augmented prediction model combining baseline scores with lesion-level stigmata and hemostasis modality. Paquet variceal grade (per 1-grade increase) was included as a candidate predictor in the endoscopy-augmented model and contributed modestly (penalized OR ≈1.10; Appendix A). Primary sources for contextual score definitions are provided in Appendix B.

**Table 6:** Discrimination for five-day rebleeding using baseline scores and an endoscopy-augmented model. Note: 95% CIs were derived by bootstrap resampling (1,000 iterations). ORs and p-values are from univariable logistic regression per 1 SD increase in baseline scores; the endoscopy-augmented model is multivariable (Appendix A). Statistical significance was predefined as *P* < 0.05 (two-sided). AUROC, area under the receiver operating characteristic curve; CI, confidence interval; CLIF-C AD, CLIF Consortium Acute Decompensation score; MELD-Na, Model for End-Stage Liver Disease with sodium; AIMS65, albumin, INR, mental status, systolic blood pressure, age ≥65 years score

Score	AUROC	95% CI	OR (95% CI)	P
CLIF-C AD	0.523	0.409-0.638	0.95 (0.66-1.38)	0.801
MELD-Na	0.552	0.447-0.656	0.85 (0.57-1.25)	0.400
AIMS65	0.549	0.443-0.654	0.85 (0.58-1.23)	0.383
Endoscopy-augmented model	0.720	0.621-0.819	-	-

In univariable logistic regression, baseline physiologic severity scores were not associated with five-day rebleeding (per 1 SD increase: CLIF-C AD OR 0.95, 95% CI 0.66-1.38, *P *= 0.801; MELD-Na OR 0.85, 95% CI 0.57-1.25, *P *= 0.400; AIMS65 OR 0.85, 95% CI 0.58-1.23, p=0.383). Endoscopic stigmata distributions (active variceal bleeding, white nipple sign, red wale mark, Other) did not differ significantly between rebleeders and non-rebleeders overall (Fisher’s exact, Monte Carlo, *P *= 0.512), whereas hemostasis modality differed (Fisher’s exact, Monte Carlo, *P *= 0.003), with higher rebleeding rates after APC (33.3%) compared with EBL (13.8%) and no rebleeding events in conservatively managed patients. These therapy associations are interpreted as non-causal and likely reflect confounding by indication (therapy selection tracking perceived lesion severity and technical difficulty). Formal pairwise DeLong comparisons for five-day rebleeding likewise showed no statistically significant differences between CLIF-C AD and MELD-Na (*P* = 0.435), CLIF-C AD and AIMS65 (*P* = 0.482), or MELD-Na and AIMS65 (*P* = 0.918).

After bootstrap optimism correction, the endoscopy-augmented model AUROC decreased to 0.671, underscoring the need for external validation.

**Figure 4:**
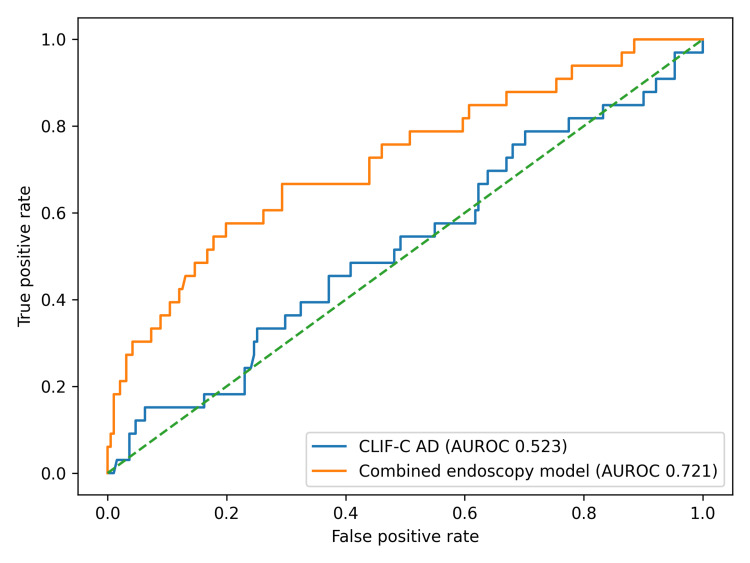
ROC curves for five-day rebleeding. Comparison of CLIF–C AD alone versus an endoscopy–augmented model (baseline scores + endoscopic stigmata + hemostasis modality). Note: The endoscopy-augmented model is intended for post-endoscopy risk stratification; associations may reflect confounding by indication (therapy selection based on lesion severity). ROC, receiver operating characteristic; AUROC, area under the ROC curve; CLIF-C AD, CLIF Consortium Acute Decompensation score; MELD-Na, Model for End-Stage Liver Disease with sodium; AIMS65, albumin, INR, mental status, systolic blood pressure, age ≥65 years score

Discrimination for rebleeding using baseline scores alone versus the endoscopy-augmented model is shown in Figure 4.

Endoscopic stigmata and hemostasis modality distributions by rebleeding status are summarized in Table 7.

**Table 7:** Endoscopic stigmata and hemostasis modality by five-day rebleeding status. Note: Endoscopic stigmata are reported as active variceal bleeding, white nipple sign, red wale marks, and others. The Forrest classification was used to map ulcer lesion stigmata (Appendix A for model coefficients and Figure 1 for calibration). *P*-values are overall tests for the multi-level variables (stigmata distribution and hemostasis modality). Because several cells were sparse, we used Fisher’s exact test with Monte Carlo simulation (10,000 replicates) for both overall comparisons. No category-level *P*-values are reported to avoid multiple unadjusted comparisons. Statistical significance was predefined as *P* < 0.05 (two-sided). EBL, endoscopic band ligation; APC, argon plasma coagulation

Variable	No rebleed (*n* = 191)	Rebleed (*n* = 33)	P
Endoscopic stigmata (highest-risk lesion)			0.512
Active variceal bleeding	113 (59.2%)	22 (66.7%)	
White nipple sign	52 (27.2%)	5 (15.2%)	
Red wale mark	18 (9.4%)	4 (12.1%)	
Other	8 (4.2%)	2 (6.1%)	
Hemostasis modality			0.003
EBL	131 (68.6%)	21 (63.6%)	
APC	20 (10.5%)	10 (30.3%)	
Balloon	1 (0.5%)	1 (3.0%)	
Conservative	32 (16.8%)	0 (0.0%)	
Other	7 (3.7%)	1 (3.0%)	

Risk gradients across CLIF-C AD strata

To translate model performance into clinically interpretable strata, Table 8 shows observed event rates across CLIF-C AD strata (as defined in the database). Mortality and ICU admission increased stepwise across strata, supporting face validity for post-bleed prognostication.

**Table 8:** Observed outcomes across CLIF-C AD strata (risk gradient). Note: Event rates shown are unadjusted. CLIF-C AD, CLIF Consortium Acute Decompensation score; ICU, intensive care unit

CLIF-C AD strata	n	ICU admission, *n* (%)	Six-week mortality, *n* (%)	Five-day rebleeding, *n* (%)
High	75	29 (38.7%)	59 (78.7%)	10 (13.3%)
Intermediate	74	5 (6.8%)	12 (16.2%)	10 (13.5%)
Low	75	3 (4.0%)	5 (6.7%)	13 (17.3%)

Observed six-week mortality increased across CLIF-C AD-predicted risk bands derived from the fitted logistic model: 7.4% for predicted risk <10%, 10.3% for 10%-19%, and 57.3% for ≥20% (Table 9).

**Table 9:** Observed six-week mortality by CLIF-C AD predicted-risk bands. Note: Predicted risks were generated from a logistic regression model with CLIF-C AD as a continuous predictor; patients were grouped into <10%, 10%-19%, and ≥20% predicted-risk bands. CLIF-C AD, CLIF Consortium Acute Decompensation score

Predicted six-week mortality risk (CLIF-C AD model)	n	Six-week deaths	Observed six-week mortality, %	In-hospital deaths	Post-discharge deaths (≤6 weeks)
<10%	68	5	7.4	3	2
10%-19%	39	4	10.3	1	3
≥20%	117	67	57.3	49	18

## Discussion

Across contemporary portal hypertension guidance, the clinically meaningful horizon for acute variceal bleeding extends beyond discharge. Six-week mortality captures the early post-bleed cascade of recurrent hemorrhage, infection, and organ failure and is therefore the preferred endpoint for prognostic work in this setting [1]. CLIF-C AD showed strong discrimination for six-week mortality and a clear risk gradient across CLIF strata, supporting its utility as a pragmatic post-bleed prognostic tool. Endoscopic variceal size (Paquet grade) was evaluated as a candidate predictor for early rebleeding given its lesion-level severity information. Because early rebleeding is heavily lesion- and procedure-dependent, incorporating a lesion-level descriptor is clinically appropriate and reduces the risk of over-interpreting global physiology scores for rebleeding. In our cohort, global scores had limited discrimination for five-day rebleeding, whereas Paquet grade contributed modestly when evaluated as a candidate predictor in the endoscopy-augmented model (penalized OR ≈1.10 per grade; Appendix A), supporting its use as a lesion-severity descriptor that may be leveraged in future rebleeding-focused models. This aligns with recommended reporting practices for observational studies and prediction models [14,15]. Limitations include the retrospective single-center design, lack of time-to-death within six weeks and time-to-rebleed within five days (precluding survival/time-to-event modeling), and limited granularity on some endoscopic and procedural details (e.g., portal pressure surrogates and timing of therapy). External validation remains necessary before applying any thresholds across centers.

Conceptually, CLIF-C AD is an appealing post-bleed tool because it was derived for hospitalized patients with acute decompensation without ACLF and incorporates systemic inflammation (leukocyte count) alongside renal and circulatory dysfunction (creatinine and sodium) and synthetic dysfunction (INR) [6,7]. Systemic inflammation and renal dysfunction are well-established drivers of short-term mortality in acutely decompensated cirrhosis and are central to the EASL-CLIF framework for severe decompensation syndromes [8,9]. Bleeding is a prototypical decompensating event, and these physiologic domains plausibly mediate the vulnerability to post-bleed deterioration even after endoscopic hemostasis. From a pragmatic standpoint, it is not surprising that MELD-Na and AIMS65 performed similarly to CLIF-C AD for six-week mortality. MELD-Na is an established cirrhosis prognostic model, and sodium improves its mortality prediction, with hyponatremia consistently associated with increased risk in cirrhosis [4,5]. AIMS65, while not cirrhosis-specific, has repeatedly shown robust performance for mortality and resource utilization in general UGIB cohorts [2]. In practice, this suggests a workflow-centric interpretation: where MELD-Na or AIMS65 are already embedded in local pathways, CLIF-C AD may add value primarily by offering an acute-decompensation framing and by providing a single score that aligns with the CLIF conceptual model.

Early rebleeding is a harder target. Five-day rebleeding is driven by lesion-level factors (variceal size and stigmata; for ulcers, Forrest classification), adequacy of endoscopic therapy, portal pressure dynamics, and early infection [1,17]. Global physiology scores, therefore, underperform compared with lesion-focused predictors and treatment variables. This was reflected in our cohort, where AUROC estimates for rebleeding were lower, and baseline severity scores were not clearly separated between rebleeders and non-rebleeders. Accordingly, our secondary endpoint analysis incorporated endoscopic stigmata and hemostasis modality to increase lesion- and procedure-level granularity for rebleeding prediction. Notably, apparent discrimination of the endoscopy-augmented model attenuated after bootstrap optimism correction (AUROC 0.671), underscoring the need for external validation before clinical deployment.

Clinical implications should be framed around decisions that occur after initial stabilization and endoscopy: ICU triage (or higher-acuity monitoring), thresholds for escalation discussions, and discharge planning with structured early follow-up for high-risk patients. In this cohort, a substantial proportion of six-week deaths occurred after discharge, supporting the clinical relevance of discharge-facing risk stratification. Guideline-based bundles remain central, including early vasoactive therapy and antibiotic prophylaxis for suspected variceal bleeding and a restrictive transfusion strategy [17,18,19,20]. CLIF-C AD could be used to support these workflow decisions by identifying patients likely to deteriorate despite endoscopic hemostasis.

This study has several strengths: complete short-term follow-up, universal urgent endoscopy under a standardized protocol, and a prespecified restriction of the primary analysis to three scores and two endpoints to limit multiplicity. Limitations include the retrospective single-center design and lack of time-to-event information for six-week mortality and for rebleeding, which precluded survival/time-to-event modeling and may limit transportability. Pragmatically, a predicted six-week mortality risk ≥20% may justify ICU-level monitoring (or step-up care) after hemostasis, while risks ≥10% may define a discharge-caution band supporting delayed discharge and structured early follow-up. These thresholds are illustrative and require external validation. The mixed-acuity composition of the cohort further supports broader applicability to routine tertiary-care admissions rather than only to patients requiring ICU-level care at presentation.

## Conclusions

Six-week mortality is a clinically meaningful endpoint for cirrhotic UGIB because it captures adverse trajectories that extend beyond the index hospitalization. In this cohort, CLIF-C AD provided strong discrimination for six-week mortality and clear risk separation across strata, performing comparably to MELD-Na and AIMS65. These results support CLIF-C AD as a pragmatic post-bleed prognostic tool when clinicians wish to frame risk in terms of acute decompensation physiology.

Prediction of five-day rebleeding using baseline physiologic scores (CLIF-C AD, MELD-Na, and AIMS65) remained modest, consistent with rebleeding being driven by lesion-level and procedural determinants rather than systemic decompensation alone. When we increased endoscopic granularity using endoscopic lesions and incorporated hemostasis modality, an endoscopy-augmented model achieved improved discrimination. This model is most appropriately interpreted as a post-endoscopy risk stratification tool to inform monitoring intensity and discharge planning, while recognizing potential confounding by indication (therapy selection reflects perceived lesion severity). Accordingly, coefficients for hemostasis-related variables should be viewed as predictive features rather than estimates of treatment effect. Granular stigmata descriptors can, therefore, complement mortality-focused scores by targeting hemostasis failure risk.

## Appendices

Appendix A

This cohort contains multiple routinely used UGIB and liver-specific scores that are frequently referenced in clinical practice and in prior UGIB-in-cirrhosis literature. We report a limited set of additional scores as descriptive context to help readers benchmark the prespecified primary comparators (CLIF-C AD, MELD-Na, AIMS65) against familiar alternatives, to facilitate cross-study comparability.

Primary sources for contextual score definitions are provided in Supplementary references (S1-S5).

We report AUROC values without CIs or hypothesis testing, and we do not interpret rank order as definitive.

**Table 10:** Contextual AUROC values for 6–week mortality across additional scores (descriptive only). Abbreviations: AUROC = area under the receiver operating characteristic curve; PTAR = prothrombin time/INR–to–albumin ratio; ALBI = albumin–bilirubin; PALBI = platelet–albumin–bilirubin; BAR = blood urea nitrogen–to–albumin ratio; GBS = Glasgow–Blatchford score; MELD = Model for End–stage Liver Disease; iMELD = integrated MELD. Note: Descriptive table only; CIs and p–values intentionally omitted.

Score	AUROC
CLIF–C AD	0.891
AIMS65	0.886
PTAR	0.882
MELD	0.879
MELD–Na	0.866
iMELD	0.864
ALBI	0.857
PALBI	0.827
BAR	0.782
GBS	0.748
Full Rockall	0.728

Appendix B: Supplementary references

S1. Blatchford O, Murray WR, Blatchford M. A risk score to predict need for treatment for upper-gastrointestinal haemorrhage. Lancet. 2000;356(9238):1318-1321. doi:10.1016/S0140-6736(00)02816-6.

S2. Rockall TA, Logan RFA, Devlin HB, Northfield TC. Risk assessment after acute upper gastrointestinal haemorrhage. Gut. 1996;38(3):316-321. doi:10.1136/gut.38.3.316.

S3. Gao F, Cai MX, Lin MT, et al. Prognostic value of international normalized ratio to albumin ratio among critically ill patients with cirrhosis. Eur J Gastroenterol Hepatol. 2019;31(7):824-831. doi:10.1097/MEG.0000000000001339.

S4. Elshaarawy O, Abdelsameea E, Abed S, et al. Platelet-albumin-bilirubin score as a predictor of outcome in acute variceal bleeding. World J Gastroenterol. 2020;26(10):1058-1070. doi:10.3748/wjg.v26.i10.1058.

S5. Johnson PJ, Berhane S, Kagebayashi C, et al. Assessment of liver function in patients with hepatocellular carcinoma: a new evidence-based approach - the ALBI grade. J Clin Oncol. 2015;33(6):550-558. doi:10.1200/JCO.2014.57.9151.
